# Lack of Involvement of CEP Adducts in TLR Activation and in Angiogenesis

**DOI:** 10.1371/journal.pone.0111472

**Published:** 2014-10-24

**Authors:** John Gounarides, Jennifer S. Cobb, Jing Zhou, Frank Cook, Xuemei Yang, Hong Yin, Erik Meredith, Chang Rao, Qian Huang, YongYao Xu, Karen Anderson, Andrea De Erkenez, Sha-Mei Liao, Maura Crowley, Natasha Buchanan, Stephen Poor, Yubin Qiu, Elizabeth Fassbender, Siyuan Shen, Amber Woolfenden, Amy Jensen, Rosemarie Cepeda, Bijan Etemad-Gilbertson, Shelby Giza, Muneto Mogi, Bruce Jaffee, Sassan Azarian

**Affiliations:** 1 Analytical Sciences, Novartis Institutes for Biomedical Research, Cambridge, MA, United States of America; 2 Global Discovery Chemistry, Novartis Institutes for Biomedical Research, Cambridge, MA, United States of America; 3 Developmental and Metabolic Pathways, Novartis Institutes for Biomedical Research, Cambridge, MA, United States of America; 4 Ophthalmology, Novartis Institutes for Biomedical Research, Cambridge, MA, United States of America; Indiana University College of Medicine, United States of America

## Abstract

Proteins that are post-translationally adducted with 2-(ω-carboxyethyl)pyrrole (CEP) have been proposed to play a pathogenic role in age-related macular degeneration, by inducing angiogenesis in a Toll Like Receptor 2 (TLR2)-dependent manner. We have investigated the involvement of CEP adducts in angiogenesis and TLR activation, to assess the therapeutic potential of inhibiting CEP adducts and TLR2 for ocular angiogenesis. As tool reagents, several CEP-adducted proteins and peptides were synthetically generated by published methodology and adduction was confirmed by NMR and LC-MS/MS analyses. Structural studies showed significant changes in secondary structure in CEP-adducted proteins but not the untreated proteins. Similar structural changes were also observed in the treated unadducted proteins, which were treated by the same adduction method except for one critical step required to form the CEP group. Thus some structural changes were unrelated to CEP groups and were artificially induced by the synthesis method. In biological studies, the CEP-adducted proteins and peptides failed to activate TLR2 in cell-based assays and in an *in vivo* TLR2-mediated retinal leukocyte infiltration model. Neither CEP adducts nor TLR agonists were able to induce angiogenesis in a tube formation assay. *In vivo*, treatment of animals with CEP-adducted protein had no effect on laser-induced choroidal neovascularization. Furthermore, *in vivo* inactivation of TLR2 by deficiency in Myeloid Differentiation factor 88 (Myd88) had no effect on abrasion-induced corneal neovascularization. Thus the CEP-TLR2 axis, which is implicated in other wound angiogenesis models, does not appear to play a pathological role in a corneal wound angiogenesis model. Collectively, our data do not support the mechanism of action of CEP adducts in TLR2-mediated angiogenesis proposed by others.

## Introduction

Age-related macular degeneration (AMD) is a major cause of legal blindness in the elderly. The macula is a specialized area of the central retina that is enriched in photoreceptor cells and is responsible for high acuity vision. In AMD, progressive macular degeneration can impair critical daily functions such as reading, driving, and face recognition. Thus AMD can have a profound impact on quality of life. There are two forms of advanced AMD: dry and wet (neovascular) AMD [1]. AMD is thought to be a disease of the retinal pigment epithelium (RPE) cells, which provide critical support functions to adjacent photoreceptors [1]. In the early stage of disease, AMD retinas show progressive accumulation of extracellular deposits, drusen, as well as intracellular deposits, lipofuscin, at the level of the RPE. These deposits initially tend to accumulate in the macular area. Over time, RPE cells show pigmentary changes and begin to degenerate. In advanced stages, dry AMD patients exhibit substantial delineated areas of RPE atrophy, or geographic atrophy. Advanced wet AMD patients exhibit leaky blood vessels in the macula, in many cases emanating from the choriocapillaris [1].

Currently there are no treatments for dry AMD. In the Age-Related Eye Disease Study 1 (AREDS 1), dietary supplements comprised of anti-oxidants and select minerals reduced the risk of progression to advanced AMD by 25% [1]. Several therapeutic approaches are being tested in clinical trials [2] but there are no FDA-approved treatments in practice at this point. For wet AMD, anti-angiogenic treatments have been clinically proven to be efficacious [3]. However, not all patients respond to treatment and the burden of treatment is still relatively high. Thus there is a great medical need for novel treatments for AMD. The molecular details of pathogenesis in AMD are not fully established but several pathogenic mechanisms have been implicated [1]. For example, human molecular genetic data indicate the involvement of the alternative complement pathway. Another potential cause is proposed to be cumulative oxidative stress, based on preclinical studies and on the AREDS1 trial. One manifestation of oxidative stress is proposed to be the formation of CEP adducts, which are a type of advanced glycation end products [4].

Photoreceptor cells are highly enriched in docosahexaenoic acid (DHA), a labile fatty acid that is susceptible to breakdown by photo-oxidation and other forms of oxidative stress. The breakdown products include a reactive aldehyde, 4-hydroxy-7-oxohept-5-enoic acid, which can condense with primary amines to form a Schiff base. In the case of proteins, 4-hydroxy-7-oxohept-5-enoic acid condenses with lysine ε-amines. Subsequent reactions result in a covalently attached CEP moity, yielding a stable CEP adduct [4]. In previous reports, antibodies raised against synthetic CEP reagents were used to identify, localize, and quantify CEP adducts by various immunological assays [5]. Elevated levels of CEP adducts were initially reported in proteomic studies of AMD donor eyes [5] and subsequently in AMD plasma [6], [7]. Thus CEP adducts were implicated in AMD [5]–[7]. In later studies CEP adducts were reported to be pro-angiogenic, both *in vitro* and *in vivo*. These *in vivo* studies utilized the micropocket corneal neovascularization (CoNV) and the laser-induced choroidal neovascularization (CNV) models [8]. More recently, Toll-like receptor 2 (TLR2) was reported to mediate the CEP adduct-induced angiogenesis [9]. The angiogenic activity was reported to be independent of the vascular endothelial growth factor (VEGF) pathway [8], [9].

There is a medical need for novel treatments for both wet and dry AMD. CEP adducts are implicated in both forms of AMD and represent an attractive potential target for drug discovery. Thus we initiated validation studies to assess the therapeutic potential of inhibiting CEP adducts.

## Results

### Synthesis of Tool Reagents

Several synthetic CEP adducts were generated according to reported procedures [10]. These tool reagents included protein (e.g. human serum albumin-CEP, or HSA-CEP), peptide (e.g. Ac-Gly-Lys-OMe-CEP, or dipeptide-CEP), and phospholipid (e.g. phosphatidyl ethanolamine-CEP, or PE-CEP) adducts, as listed in **Table S1**. The presence of CEP adducts and the stoichiometry of adduction was confirmed by ^1^H-NMR and LC-MS/MS (**Figure S1 and Figure S2**). Invariably the presence of CEP moiety was established in the adducted samples and was never detected in the controls. CEP adduction was deemed successful by several measures. For example, ^1^H-NMR analysis indicated the expected molecular signature of the CEP group in dipeptide-CEP but not the untreated dipeptide (**Figure S1B**). Likewise, LC-MS/MS analysis of enzymatic hydrolyzed adducted proteins detected lysine-CEP in HSA-CEP and MSA-CEP (mouse serum albumin-CEP) but not the respective controls (**Figure S1C**). For protein adducts, two controls were used: CTL1, which represents untreated protein; and CTL2, which represents treated unadducted protein. The latter control was treated exactly the same way as the corresponding CEP adduct except for one step, to prevent adduction (see Materials and Methods). No CEP moiety was detected in HSA-CTL2 or MSA-CTL2. There are a total of 59 lysines in HSA (UnitPro P02768) and 50 lysines in MSA (UnitPro P07724). We identified 14 lysine-CEP sites in HSA-CEP and 40 lysine-CEP sites in MSA-CEP (**Figure S2**). These values are in fair agreement with the reported stoichiometries: 6 or 17 CEP-modified lysines for HSA-CEP [10], [11], and 15 for MSA-CEP [10].

### Does CEP-Adduction Affect Protein Structure?

**Figure 1:**
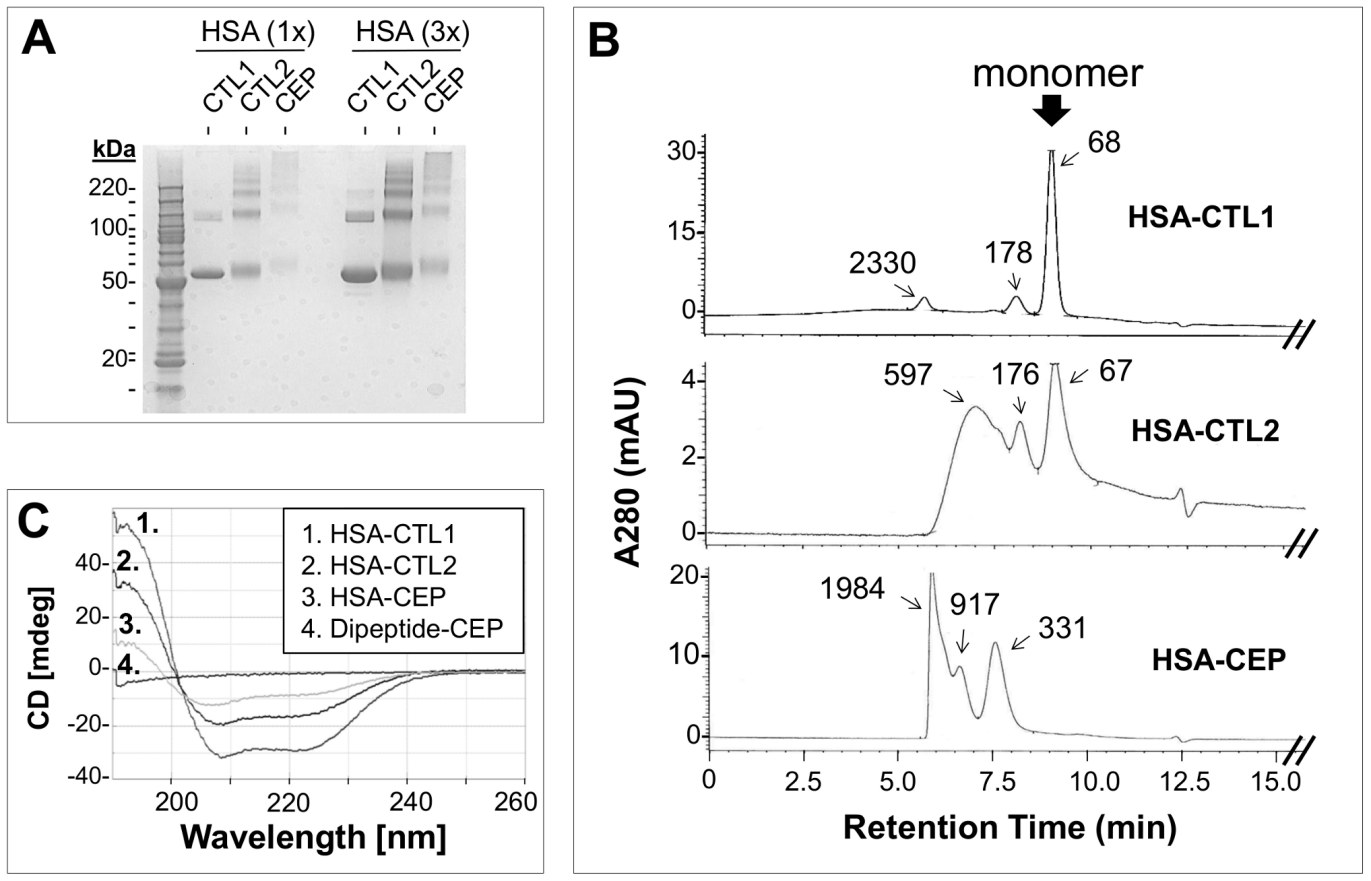
Structural Analyses of CEP Adducts. A) *SDS-PAGE analysis*. Aliquots of HSA-CTL1 (untreated), HSA-CTL2 (treated but unadducted) and HSA-CEP were subjected to reducing SDS-PAGE on 4–10% gels. Compared to HSA-CTL1, both HSA-CTL2 and HSA-CEP showed an increase in high-MW bands. B) *Size exclusion chromatography*. SEC under non-denaturing conditions indicated an increase in faster-eluting peaks in HSA-CTL2 and HSA-CEP compared to HSA-CTL1. C) *Circular dichroism*. CD analysis revealed a loss of secondary structure in HSA-CTL2 and HSA-CEP compared to HSA-CTL1.

When we analyzed HSA-CEP, HSA-CTL2, and HSA-CTL1 in structural studies we observed significant structural alterations in HSA-CEP in comparison with HSA-CTL1. However, HSA-CTL2 also incurred significant alterations, similar to but less extensive than HSA-CEP. On SDS-PAGE gels HSA-CEP and HSA-CTL2 but not HSA-CTL1 appeared to form oligomers in a ladder-like fashion (**Figure 1A**). Comparison of size exclusion chromatography (SEC) profiles indicated progressive loss of the HSA monomer in the order of HSA-CTL1> HSA-CTL2> HSA-CEP (**Figure 1B**). Circular dichroism (CD) also indicated a significant loss in secondary structure in HSA-CTL2 and HSA-CEP compared to HSA-CTL1 (**Figure 1C**).

Collectively these data show that our synthetic CEP adducts can incur two kinds of structural changes: a) CEP-independent changes and b) CEP-dependent changes. Comparison of our HSA-CTL1 and HSA-CTL2 illustrates the CEP-independent changes in protein structure, which indicate artificial changes introduced by the procedure for generating synthetic adducts. Comparison of our HSA-CTL2 and HSA-CEP illustrates the CEP-dependent changes that occur as a result covalent CEP groups in HSA-CEP, beyond the CEP-independent changes in HSA-CTL2.

### Is TLR2 Activated By CEP Adducts?

**Figure 2:**
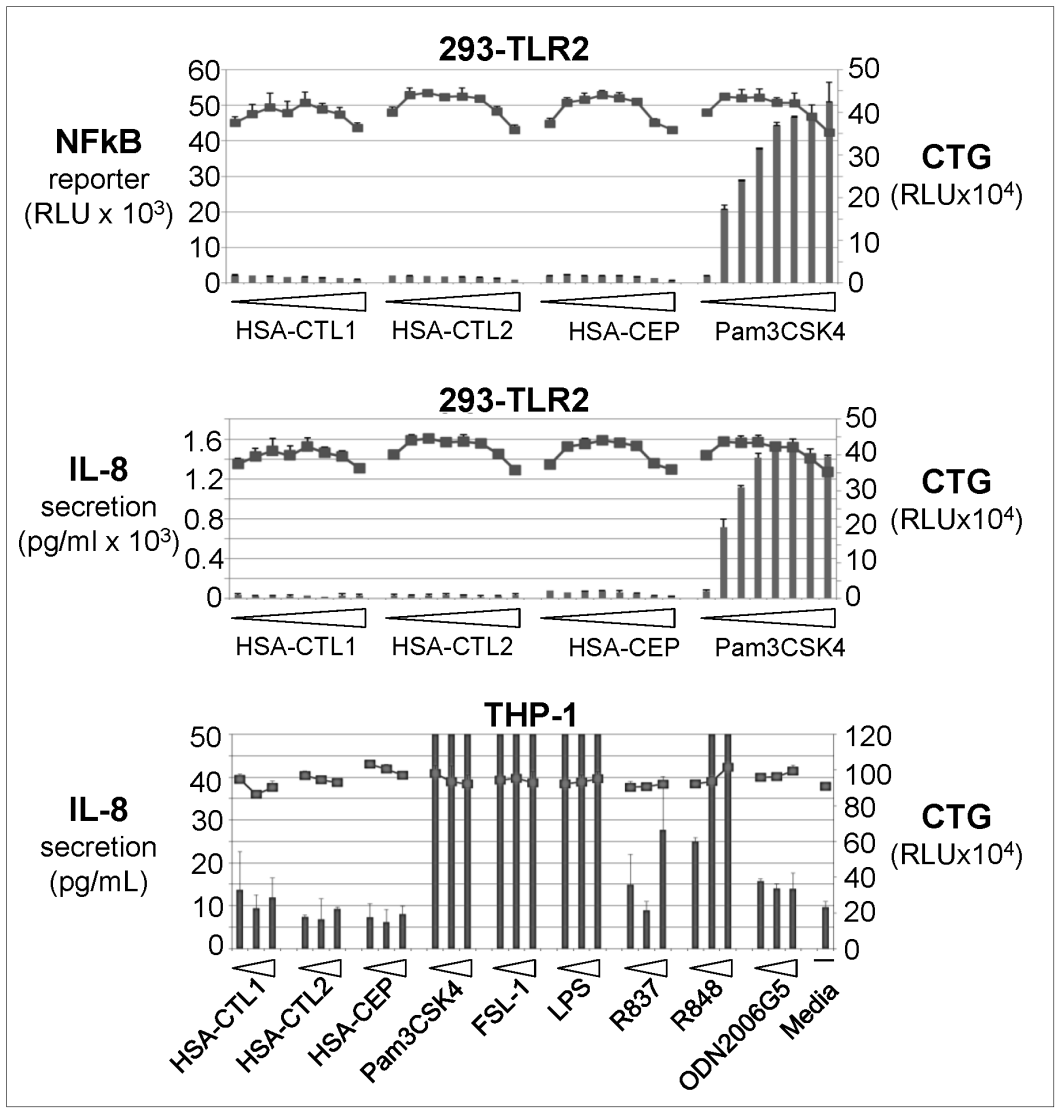
Cell-Based TLR Activation Assays. Various CEP adducts and TLR agonists were tested in HEK293 or THP-1 cells. Readouts were NFkB reporter signal or IL-8 secretion (columns), as indicated. In addition, the same wells were analyzed for viability with the CellTiter-Glo kit (axis on right; square symbols) to ensure that any lack of activation was not due to cell toxicity. HEK293 cells were treated with the following reagents: HSA-CTL1, HSA-CTL2, or HSA-CEP: 0, 3.9, 7.8, 15.6, 32.6, 62.5, and 125 and 250 µg/ml; Pam3CSK4∶ 0, 1.5, 3.2, 6.3, 12.5, 25, 50, 100 ng/mL. THP-1 cells were treated with the following reagents: HSA-CTL1, HSA-CTL2, or HSA-CEP: 62.5, 125, and 250 µg/mL; Pam3CSK4∶ 4, 20, and 100 ng/mL; FSL-1∶0.4, 2, and 10 ng/mL; LPS: 4, 20, and 100 ng/mL; R837 or R848∶0.4, 2, and 10 µM; ODN2006G5∶0.2, 1, and 5 µM.

**Figure 3:**
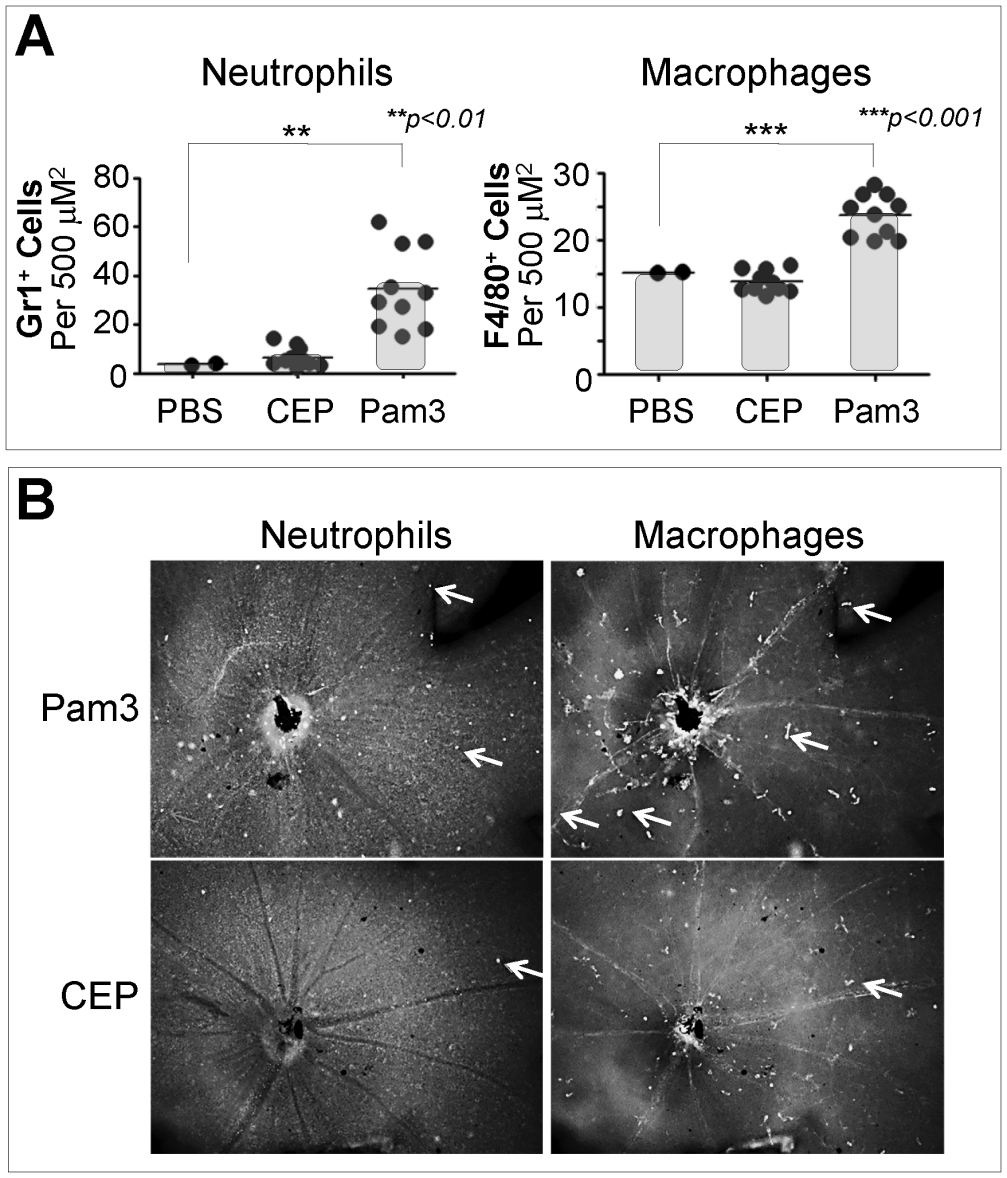
Retinal Leukocyte Infiltration Assay. A) Mice were injected intraperitoneally with either PBS, Pam3CSK4 (25 µg per animal, in PBS), or dipeptide-CEP (400 µg per animal, in PBS) and the retinas were analyzed 8 hours later. Retinal infiltration by neutrophils (Gr1+ cells) or macrophages (F4/80+ cells) was assessed by immunostaining with the respective markers and quantitated with Axiovision, as described in Materials and Methods. Statistical analysis was performed using the Student t-test. Only statistically significant differences are indicated in the graph. B) Shown are representative images of the experiment in **Figure 3A**. Arrows indicate examples of macrophages or neutrophils in the corresponding images. *CEP*, Dipeptide-CEP; *Pam3*, Pam3CSK4.

We tested several CEP adducts in a cell-based TLR2 assay, essentially as described [9]. Pam3CSK4, a known TLR2 agonist, showed dose-dependent activation of TLR2 as monitored by production of NF-κB or IL-8 in cellular assays (**Figure 2**, top and middle panels). However, no TLR2 activation was detected by HSA-CEP (**Figure 2**), or several other CEP adducts tested: MSA-CEP, dipeptide-CEP, or PE-CEP (not shown). None of these adducts showed any cytotoxicity, as measured with CellTiter-Glo (CTG) kit (**Figure 2**). Next we used THP-1 cells, which naturally express several TLRs, including TLR2. While positive controls showed specific activation of the corresponding TLR, CEP adducts failed to activate TLR2 or any other TLR that was monitored (**Figure 2** **,** bottom panel). *In vivo*, CEP adducts did not induce biological effects that are mediated by TLR2. As **Figure 3A** shows, treatment of mice with Pam3CSK4 induced infiltration of neutrophils and macrophages in the retina. However, neither dipeptide-CEP nor MSA-CEP (not shown) induced retinal infiltration in the same assay. Representative images of this experiment are shown in **Figure 3B**. These results suggest that CEP adducts do not activate TLRs, including TLR2.

### Are CEP Adducts or TLR2 Involved in Angiogenesis?

**Figure 4:**
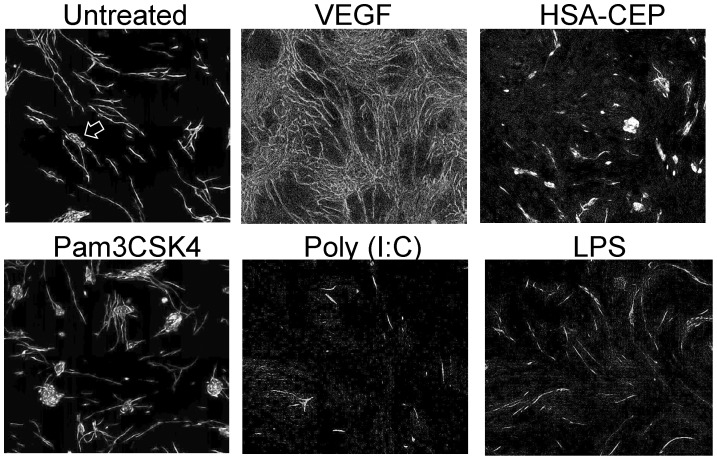
Tube Formation Assay. Shown are representative images of the experiments in presented numerically in **Table 1** **.** The figure shows images for untreated negative control (untreated), positive control (VEGF 165, 4 ng/mL), HSA-CEP (2 µg/mL), Pam3CSK4 (500 nM), LPS (10 ng/mL), poly (I:C) (10 µg/mL). The arrow in the untreated image shows an example of an island of unmigrated HUVEC cells, which is also seen in other images.

We used the tube formation *in vitro* assay to determine if CEP adducts are angiogenic, similar to a previously reported assay [9]. VEGF induced significant tube formation; however, neither CEP adducts nor Pam3CSK4 affected tube formation (**Table 1**). In addition, poly (I:C) (a TLR3 agonist) and LPS (a TLR4 agonist) failed to show any effect. Representative images of the tube formation assay are shown in **Figure 4**.

**Table 1:** Evaluation of CEP Adducts and TLR Agonists in the Tube Formation Assay.

	Reagent	Concentration	Average Tube Length (mm/mm^2^)	Std Dev	P value^a^
**Experiment 1**	untreated	–	1.97	+/−0.35	–
	LPS	10 ng/mL	3.38	+/−0.59	0.0072
	Poly (I:C)	10 µg/mL	0.16	+/−0.09	0.0005
	Pam3CSK4	500 nM	2.13	+/−0.24	0.9997
	VEGF^b^	3.1 ng/mL	10.61	–	<0.0001
**Experiment 2**	untreated	–	1.85	+/−0.27	–
	HSA-CEP	2 µg/mL	1.18	+/−0.32	0.1079
	HSA-CTL2	2 µg/mL	2.32	+/−0.45	0.3594
	VEGF	4 ng/mL	9.00	+/−1.64	<0.0001

GFP-transfected HUVEC, co-cultured with human fibroblasts, were treated on days 1, 2, 5, 7 and 9 with VEGF, TLR agonists, HSA-CEP, or HSA-CTL2. Control wells (“untreated”) received media alone. Average tube length (mm/mm^2^) was determined by fluorescence measurements as described in Materials and Methods. Representative images from these two experiments are shown in **Figure 3**.

a One way ANOVA with Dunnett's multiple comparison test, compared to untreated sample.

b This VEGF control was measured in duplicate and not in triplicate, therefore no Std Dev is presented.

**Figure 5:**
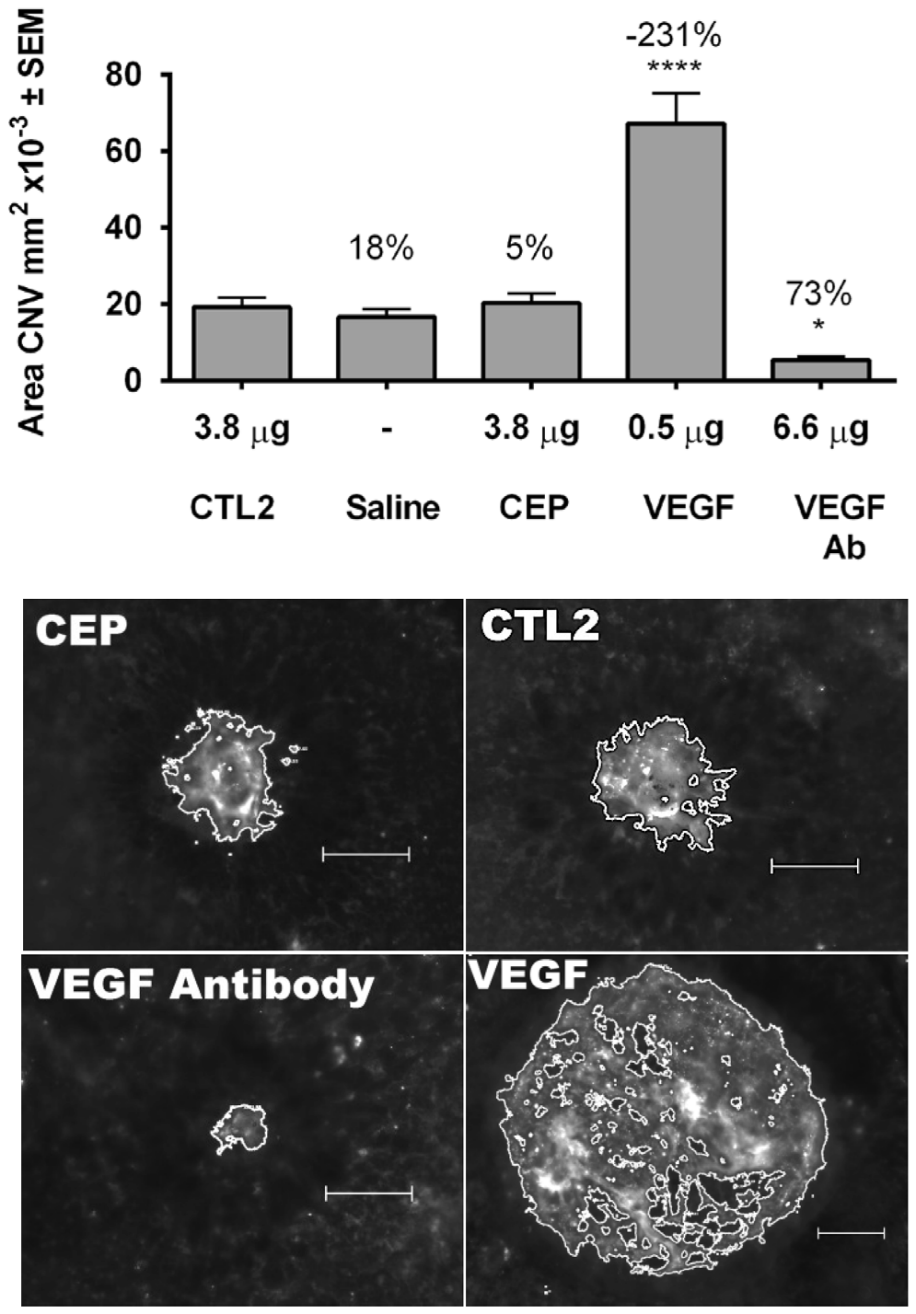
Mouse Laser-Induced CNV Assay. (A) Subretinal injection of MSA-CEP does not increase CNV area compared to mice injected with saline or MSA-CTL2. Bar graph shows mean area of CNV +/− SEM from first experiment evaluating the effect of subretinal injection of saline, 0.5 µg of rhVEGF165, 3.8 µg of MSA-CTL2, 3.8 µg of MSA-CEP, or 6.6 µg of 4G3 (an anti-mVEGF antibody) on laser-induced CNV in C57BL/6J mice. The number above each bar is the percentage inhibition relative to average CNV area in mice injected with MSA-CTL2. Subretinal injection of VEGF increases CNV area and subretinal injection of an anti-mVEGF antibody inhibits CNV area. * p<0.05, **** p<0.0001 by ANOVA with a Dunnett’s post hoc analysis. (B) Representative fluorescent images of CNV lesions 7 days after laser from mice injected in the subretinal space with MSA-CEP, MSA-CTL2, VEGF or a VEGF Antibody as described above. Scale bar = 100 microns. *CTL2*, MSA-CTL2; *CEP*, MSA-CEP.

The CEP adducts were further evaluated in the laser-induced choroidal neovascularization (CNV) model, as was reported earlier [8]. Initial laser CNV studies were performed with C57BL/6N mice, which showed no effect of MSA-CEP (**File S1**). In light of the *rd8* mutation in the *Crb1* gene reported in this strain [12], we repeated the study with C57BL/6J mice which are wildtype for *Crb1* [12] and were used in the previously reported study [8]. We observed the same results with 2 experiments in each strain. Subretinal injection of VEGF significantly exacerbated CNV, while a VEGF-neutralizing antibody inhibited CNV (**Figure 5A** **and File S1**). This is consistent with VEGF being a major pro-angiogenic factor in CNV. However, subretinally administered MSA-CEP, at a dose nearly identical to that used in the previous report [8], had no effect in this model (**Figure 5A** **and File S1**). Representative images of the experiments in Figure 5A are shown in **Figure 5B**.

**Figure 6:**
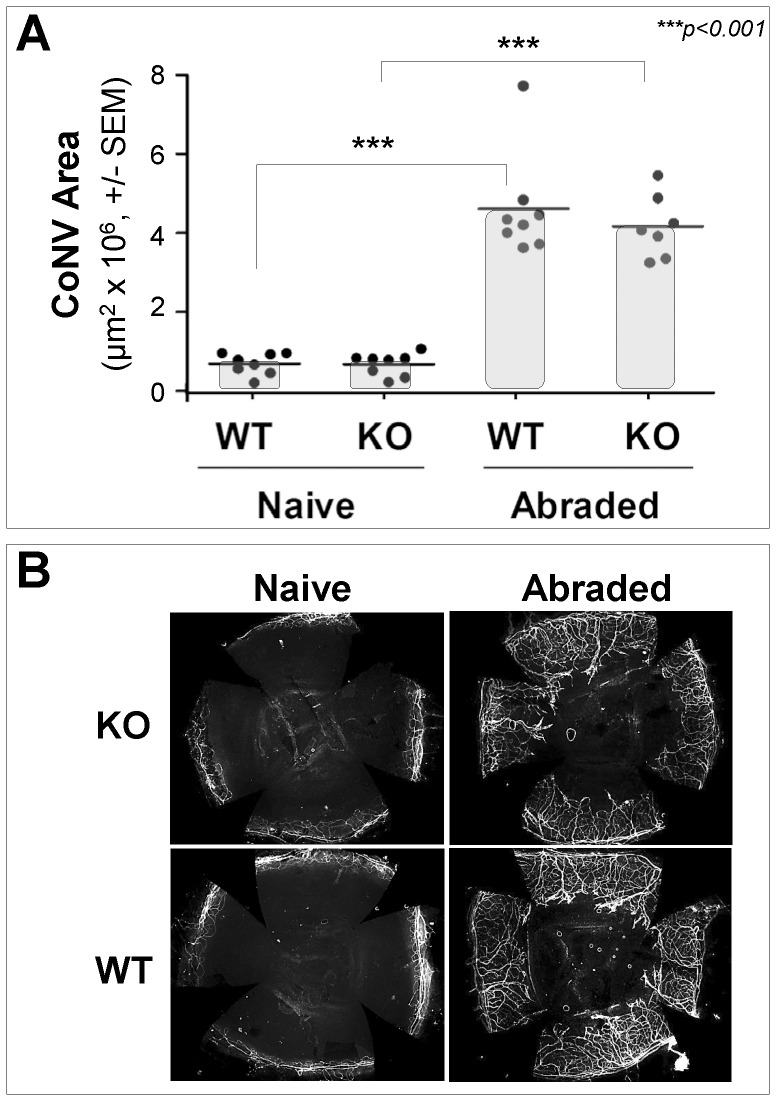
Mouse CoNV Assay. A) Adult *Myd88−/−* (KO) and littermate *Myd88+/+* (WT) mice (N = 7 to 8 animals/group) were subjected to corneal abrasion on Day 0. On Day 21 post-abrasion animals were euthanized and CoNV area (+/− SEM) was measured by fluorescence microscopy, as described in Materials and Methods. Statistical analysis was performed using two-way ANOVA. Within each genotype, the abraded group was significantly different from the naïve group (p<0.0001). However, comparison between the two genotypes showed no significant effect of the *Myd88* deficiency on CoNV area in response to abrasion. B) Representative images of the 4 groups shown in **Figure 6A**.

In studies with a corneal neovascularization (CoNV) mouse model, we observed that *Myd88*-deficiency had no significant effect on CoNV (**Figure 6**) when compared with similarly treated wild-type mice. Since *Myd88* deficiency abolishes TLR2 activity, this indicates that TLR2 is not required for angiogenesis in the abrasion-induced CoNV model. In contrast, CoNV is greatly dependent on VEGF-A: a) qPCR analysis showed a 30-fold increase in *VEGF-A* mRNA expression and b) treatment of abraded mice with VEGF-A neutralizing antibody showed significant reduction in CoNV area (**Figure S3**).

## Discussion

### Summary

Here we performed validation studies for the proposed CEP-TLR2 axis to assess the therapeutic potential for wet AMD treatment. Following a published procedure we generated synthetic CEP adducts and confirmed the presence of covalent CEP groups. Structural analyses of a CEP adduct indicated changes in tertiary structure that were not observed in the naïve protein; however, similar structural changes were observed in the treated, unadducted control. Thus the physiological relevance of the observed structural changes is uncertain. Next we attempted to reproduce some of the reported biological effects of synthetic CEP adducts. When we tested our synthetic CEP adducts in *in vitro* and *in vivo* assays, we observed neither TLR2 activation nor pro-angiogenic activity. We conclude that our data do not support the CEP-TLR2 hypothesis.

### Structural Changes in CEP Adducts

In our hands the published protocol for generating CEP adducts worked successfully, by the criterion of the presence of covalently-linked CEP groups in the adduct. This protocol worked robustly with all classes of reagents tested, including proteins, dipeptide, and lipid. Furthermore, the stoichiometry of adduction of each reagent was in fair agreement with the corresponding published stoichiometry.

In our attempt to understand the biological consequences of CEP adduction we initiated protein structural studies with HSA-CEP. Since the conversion of a significant number of positively-charged lysine side chains to negatively charged CEP groups (from the carboxylate group) would greatly alter its surface electrostatic potential, we anticipated and indeed observed structural changes in HSA-CEP in comparison to untreated HSA control (HSA-CTL1). We were surprised, however, to detect similar changes in HSA-CTL2, the treated unadducted control (**Figure 1**). Our interpretation is that two kinds of structural alterations can occur in synthetic CEP adducts: a) alterations that do not involve the CEP group and were observed when comparing HSA-CTL2 to HSA-CTL1; and b) alterations that occur as a result of covalently-linked CEP groups and were observed when comparing HSA-CEP to HSA-CTL2. It is not clear which step(s) or reagents in the published adduction procedure led to the CEP-independent changes in HSA-CTL2. A candidate culprit is the organic solvent, dimethylformamide; organic solvents are known to affect the structure of some proteins. The adduction procedure entails exposure of protein to 30% dimethylformamide/PBS solution for 4 days at 37°C [10]. In searching the literature we found similar significant structural alteration in a CEP adduct published by another laboratory. A synthetic MSA-CEP adduct appeared to migrate as a continuous smear on denaturing SDS-PAGE and immunoblot, whereas the untreated MSA (the equivalent of MSA-CTL1) migrated as one predominant electrophoretic band (Figure 1 in [13]). A treated unadducted control, the equivalent of our MSA-CTL2, was not included in the report [13]. However since we used similar procedures for generating CEP adducts, in all likelihood the published MSA-CEP incorporated both CEP-independent and -dependent changes.

Thus far, no endogenous CEP adducts have been isolated directly from any biological sources and none have been characterized in the literature. For example, the stoichiometry of CEP adduction (moles CEP per mole protein) and structural properties have not been reported for any endogenous CEP adducts. Hence at this point it would not be possible to verify that any synthetic CEP adduct is representative of endogenous ones with respect to protein structure. This caveat notwithstanding, we proceeded with biological studies to see if we could reproduce the biological effects of CEP adducts with regards to TLR2 activation and angiogenesis. In our approach we included HSA-CTL2 and MSA-CTL2 in the biological assays of the corresponding CEP adducts, so we might discern biological effects that are specific to the CEP group.

### TLR2 Activation by CEP Adducts

We tested CEP-adducted protein or dipeptide in two cell-based assays: a) HEK293-TLR2 cells that specifically expressed TLR2 and, b) THP-1 cells that express multiple TLRs, including TLR2. In both assays, TLR2 activation was observed with a synthetic TLR2 agonist, Pam3CSK4, but not with synthetic CEP adducts. Specifically, we did not detect any effect of HSA-CEP in TLR2-expressing HEK293 cells, as was reported (Figure S14 in [9]). Not surprisingly the controls for our CEP adducts did not have any effects, either. Our cellular assays also did not register any effect of the dipeptide-CEP, which was reported to be pro-angiogenic in several cellular and *in vivo* assays in a TLR2-dependent manner [9]. However, this dipeptide-CEP was not tested in the same cell-based TLR assay used for HSA-CEP [9], so a direct comparison with our cell-based data is not possible.

We also evaluated CEP adducts in an *in vivo* model for TLR2 activation. In this model, treatment with Pam3CSK4 induced retinal leukocyte infiltration in wild-type mice, but not in *Myd88−/−* nor *TLR2−/−* mice (not shown). However, treatment of wild-type mice with CEP adducts did not result in measurable retinal leukocyte infiltration, indicating that TLR2 was not activated by CEP adducts *in vivo*.

### CEP-TLR2 in Angiogenesis Assays

*In vitro,* neither HSA-CEP nor Pam3CSK4 (TLR2 agonist) showed any pro-angiogenic effect in the tube formation assay with human umbilical vein endothelial cells (HUVECs). Likewise, agonists to other TLRs (LPS, poly (I:C)) were not pro-angiogenic, whereas VEGF was.

*In vivo*, synthetic MSA-CEP did not exacerbate laser-induced CNV in a mouse model as reported [8]. This was the case with two substrains of C57BL/6 mice. In the initial two laser CNV studies we used C57BL/6N mice. Subsequently, it was reported that this substrain carries the *rd8* mutation in the *Crb1* gene [12]. We then performed two additional laser CNV studies with the C57BL/6J substrain, which has the wild-type *Crb1* gene [12]. The C57BL/6J substrain is the same one used in the previously published laser CNV study [8]. We observed similar results in all four laser CNV studies: CNV was exacerbated with exogenous VEGF, ameliorated with a VEGF-neutralizing antibody, and unaffected with MSA-CEP. Collectively, these data are consistent with a major angiogenic role for the VEGF pathway, but not for CEP adducts, in the laser-induced CNV model.

As an alternative *in vivo* model of ocular angiogenesis for exploring the CEP-TLR2 hypothesis, we used an abrasion-induced corneal neovascularization model (CoNV). This CoNV model is different from that used earlier with CEP adducts: in the corneal pocket CoNV model, a pellet containing synthetic HSA-CEP was implanted in the cornea [8]. Since our synthetic CEP adducts were biologically inactive in all assays so far, we aimed to use a model where endogenous -not synthetic- CEP adducts might play a role. In the abrasion-induced CoNV model, angiogenesis occurs as part of the wound healing process, induced by mechanical abrasion of the cornea. Furthermore, it has been shown that macrophages are recruited to the cornea during early stages of neovascularization [14]. This resembles the back punch model, in which wound angiogenesis entails recruitment of macrophages [9]. CEP adducts were reported to be transiently present during this time, detected by immunocytochemistry with an antibody against synthetic CEP adducts. By immunolabeling, a substantial portion of CEP adducts was present in the recruited F4/80+ macrophages [9]. Treatment with dipeptide-CEP in this model accelerated wound closure and vascularization in a TLR2-dependent manner, as shown by the comparison of *TLR2−/−* and *TLR2+/+* mice. In the same vein, we used the abrasion-induced CoNV model and compared littermate *Myd88−/−* and *Myd88+/+* mice. (Myd88 is required for TLR2 function.) We found no difference in CoNV area between the two groups. This indicates that, in the abrasion-induced CoNV model, TLR2 and other Myd88-dependent TLRs are not involved in angiogenesis. It is not known whether endogenous CEP adducts are present in the abrasion-induced CoNV model. We also tested topical treatment with synthetic HSA-CEP, but observed no effect on CoNV (not shown). It seems therefore the CEP-TLR2 axis proposed for other wound angiogenesis models [9] does not apply to corneal abrasion-induced wound angiogenesis model tested here.

### Synthetic vs. Endogenous CEP Adducts

It has been proposed that the biological effects of CEP adducts depend solely on the presence of CEP groups and not the host carrier [9]. This was not the case in our study: neither protein CEP adducts nor dipeptide CEP adducts produced any of the published biological effects [8], [9] that were tested here. Our synthetic reagents were verified for the presence of covalently-attached CEP groups. There is no obvious explanation for these discrepancies. As innate immune receptors, TLRs recognize structures and patterns so it is conceivable that changes in structure, even if artificial, could elicit TLR activation. Thus one possibility is that there are structural differences between our synthetic CEP adducts and those used in previous studies, as the reagents were prepared at different laboratories.

So far endogenous CEP adducts from any biological systems have not been isolated and therefore none have been characterized in structural studies. Furthermore, the abnormal electrophoretic patterns seen with synthetic CEP adducts reported here or by others [13] do not seem to resemble those of *in vivo* CEP adducts detected on immunoblots, reported either in human donor material (e.g. Figure 3 in [5]) or in the light-induced rat retinal degeneration model (e.g. Figure 1 in [15]). It is therefore uncertain which synthetic CEP adducts are representative of endogenous ones; perhaps none. The final answer awaits the isolation of endogenous CEP adducts and their characterization.

By extension, the biological effects reported with synthetic CEP adducts also need to be confirmed with endogenous CEP adducts. Our HSA-CTL2 did not show any biological activity, but neither did our HSA-CEP; hence in our study there was no concern about non-physiological biological effects, which HSA-CTL2 was intended for as a control. However, a treated unadducted control would be critically important when biological activity with a synthetic CEP adduct is observed. This is exemplified in a recent publication where the unadducted control, “sham-MSA”, showed biological activity in some assays. In BALB/c macrophages, sham-MSA induced the upregulation of M1 markers and of an inflammatory gene, *KC*, 2 to 5 fold above that of control levels (Figures 1A and S2, respectively, in [16]). In some cases the sham-MSA effect represented 30%–40% in magnitude of the MSA-CEP effect: (*IL-1β*, *TNFα*, and *KC*), despite the absence of CEP groups in sham-MSA [16].

Treated unadducted controls were not used in earlier studies that reported a role for synthetic CEP adducts in TLR2 activation and in angiogenesis [8], [9]. If it is possible that our CEP adducts are different from those used by others, it is also possible that our treated unadducted controls are different. The fact that our treated, unadducted controls (HSA-CTL2, MSA-CTL2) were biologically inactive does not necessarily apply to other studies in literature, as the example above illustrates. Thus the physiological relevance of synthetic CEP adducts is unclear, especially when untreated proteins were used as the only controls in the biological assays.

Polyclonal and monoclonal antibodies raised to synthetic CEP adducts have been reported to immunolabel biological samples from AMD patients in Western blots, immunohistochemical sections, and ELISAs [5]–[7], [11]. The same antibodies reportedly immunolabelled biological samples in animal studies, e.g. [15], [17]. However, the immunolabelled proteins were not confirmed to have any CEP moieties, by other independent assays that do not use antibodies (e.g. LC-MS/MS); i.e. the immunolabelled proteins were not confirmed to be bona fide CEP adducts. For example, several candidate CEP adducts were immunolabelled on Western blots of patient donor material and subsequently identified by LC-MS/MS analysis, however the presence of covalently-linked CEP groups was not confirmed by LC-MS/MS or other assays [5]. The fact that a synthetic CEP adduct (used a control) was immunolabelled on the same western blot is not a surprise, as CEP antibodies were raised against a synthetic CEP adduct. At this point, the antibodies against synthetic CEP adducts [5] have not yet been validated for detection of endogenous CEP adducts. This is underscored by the electrophoretic changes in synthetic CEP adducts that do not seem to resemble those of endogenous CEP adducts, as explained above. Data generated by other (non-immunological) assays is needed to validate CEP antibodies.

Arguably the *in vivo* existence of CEP adducts requires confirmation, as well, since all evidence so far has been generated with these antibodies. For example, a proteomics study of AMD patient samples identified and quantified hundreds of proteins by LC-MS/MS [18]. Yet the same study reported that CEP adducts were below detection limits and “none were reliably identified” (supplemental information in [18]). On a promising note, an improved LC-MS/MS assay has been reported, with a sensitivity of 1 pmol or less of CEP-lysine in enzymatically-digested patient plasma samples [19]. According to ELISA data [7] the average levels of CEP adducts in AMD plasma is 37 pmol/mL, thus hopefully the improved LC-MS/MS assay can confirm the presence of CEP adducts *in vivo*.

In conclusion, our studies of synthetic CEP adducts did not validate the CEP-TLR2 axis in angiogenesis as proposed [8], [9]. While the cause of the discrepancies is not clear, it does seem clear that the mere presence of CEP groups is not sufficient to elicit the reported biological responses. More data, ideally with endogenous CEP adducts, is needed to understand what properties of CEP adducts, if any, can lead to TLR2 activation and to angiogenesis. In light of the prevalence of AMD and the unmet medical needs, more research into the pathophysiology of CEP adducts is warranted.

As a postscript, after submission of our manuscript an independent report [20] also showed that CEP adducts alone do not induce TLR2 signalling nor related biological effects (e.g. Figures 1A and 1B), as we have reported here. Rather, the report claims that CEP adducts potentiate the effect of a synthetic TLR2 agonist, Pam3CSK4, in cultured murine bone-marrow derived macrophages [20].

## Materials and Methods

### Synthesis and Verification of CEP Adducts

#### Synthesis

All CEP adducts were synthesized as described [10]. The dipeptide, Ac-Gly-Lys-OMe, was obtained from BACHEM; HSA from AlbuminBio; MSA from AlbuminBio or Sigma; phosphatidyl ethanolamine was from Sigma. Controls for protein CEP adducts included untreated protein (CTL1) and treated unadducted protein (CTL2). The latter control was processed in the same synthesis procedure as that for CEP adducts, except 4,7-dioxoheptanoic acid 9-fluorenylmethyl ester was left out to avoid the covalent addition of CEP moiety. Protein CEP adducts, after final dialysis in PBS, were quantified by the Bradford assay and tested for endotoxins with the Endosafe-PTS kit (Charles River). For storage, samples were filtered through 0.2 µm, divided aseptically in 1-mL aliquots, and stored at −80 C.

#### Amino acids complete digestion

Enzymatic hydrolysis was adapted from [21]. An aliquot of 100 µg of protein is dissolved in 25 µl of PBS buffer (pH 7.4). Pronase E (Sigma, Cat. # P5147) (2 mg/ml in 10 mM potassium phosphate buffer, pH 7.4, 5 µl) was added. The sample was incubated at 37°C for 24 hours. Prolidase (Sigma, Cat. # P6675) and aminopeptidase (Sigma, Cat. # A8200) (both 2 mg/ml in 10 mM potassium phosphate buffer, pH 7.4, 5 µl) were added. The sample was incubated at 37°C for 48 hours. Amino acids from enzymatic hydrolysate (10 µl) was derivatized by Waters AccQ•Fluor (Waters, Cat.# WAT052880). Then 2 µl of derivatized samples was analyzed by Xevo-G2QTOF with Waters BEH C18 2.1 × 50 mm 1.7 µm column at 50°C at 1.0 mL/min, 0.1% formic acid in water, 0.04% formic acid in acetonitrile, 3–98% B in 9 min. An aliquot of 100 µg of protein is dissolved in 25 µl of PBS buffer (pH 7.4). Pronase E (Sigma, Cat. # P5147) (2 mg/ml in 10 mM potassium phosphate buffer, pH 7.4, 5 µl) was added. The sample was incubated at 37°C for 24 hours. Aminopeptidase (Sigma, Cat. # P6675) and prolidase (Sigma, Cat. # A9934) (both 2 mg/ml in 10 mM potassium phosphate buffer, pH 7.4, 5 µl) were added. The sample was incubated at 37°C for 48 hours. Amino acids from enzymatic hydrolysate (10 µl) was derivatized by Wasters AccQ Fluor (Waters, Cat.# Wat052880). Then 2 µl of derivatized samples was analyzed by Xevo-G2QTOF with Waters BEH C18 2.1 × 50 mm 1.7 µm column at 50°C at 1.0 mL/min, 0.1% formic acid in water, 0.04% formic acid in acetonitrile, 3–98% B in 9 min.

#### NMR of protein hydrolysate samples

Hydrolyzed protein samples were prepared for NMR analysis by the addition of 5 µL of D2O (CIL) to 15 µL of Hydrolysate solutions. Sodium 3-trimethylsilyl [2,2,3,3-d4]propionate (TMSP) as added as an internal chemical shift and quantitation reference. High-resolution ^1^H-NMR spectra were acquired at 300±1 K, using a standard (D-90°-acquire) pulse sequences on a Bruker-600 Avance spectrometer (^1^H frequency of 600.26 MHz). ^1^H-NMR spectra were acquired with 256 free induction decays, 65,536 complex data points, a spectral width of 7.2 kHz, and a relaxation delay of 5 s. All spectra were processed by multiplying the FID by an exponential weighting function corresponding to a line broadening of 0.3 Hz. The CEP pyrrole resonances at ^1^H_δ_ 6.8 ppm, ^1^H_δ_ 6.1 ppm and ^1^H_δ_ 5.9 ppm were integrated relative to the aromatic resonance of phenylalanine and tyrosine using the ACD 10.0 package (Advanced Chemistry Development, Toronto, Canada).

#### LC-MS/MS confirmation of CEP-lysine adduct

Carboxyethylpyrrole (CEP) adduct presence in CEP-conjugated murine serum albumin (MSA) and human serum albumin (HSA) has been confirmed by LC-MS/MS methodologies. CEP-MSA and CEP-HSA were hydrolyzed using protease cocktails (descriptions in above, AA complete digestion), followed by Accq-Tag Ultra derivatization (Catalog number 186003836, Waters Corporation, Milford, MA). Accq-Tagged CEP-lysine ionizes in electrospray positive mode and gives a protonated molecular ion of 439.1981, which can fragment and gives a characteristic 171.1 daughter ion from the Accq-Tag and a daughter ion of 206.1 from the carboxyethylpyrrole moiety. We employed multiple reaction monitoring, 206.1 precursor scan on a Triple Quadrupole mass spectrometer. Strong signal of 439.2 → 171.1 and 439.2 → 206.1 were observed using AB Sciex API4000 Triple Quad. Precursor ion of 439.2 was observed for 206.1 daughter ion in a precursor ion scan on the same API4000. Waters Xevo G2 Q-TOF mass spectrometer was employed for its high resolution power to further confirm the presence of CEP-lysine adducts. The molecular species of 439.1981 was observed in MS scan with 5 ppm mass accuracy across the chromatographic peak; the daughter ion 206.1181 was observed in the MS/MS scan with 10 ppm mass accuracy in the MSE approach.

### Peptide Mapping for CEP Modification Location

#### Sample preparation

All solvents (HPLC grade) and chemicals were purchased from Sigma-Aldrich (St. Louis, MO) unless otherwise stated. MSA-CEP and MSA-CTL2 (50 ug each) were denatured with 6 M guanidine hydrochloride (GuHCl), reduced with 25 mM dithiothreitol (DTT), alkylated with 50 mM iodoacetamide, dialyzed against 50 mM ammonium bicarbonate using 10 kDa MWCO Slide-A-Lyzer cassettes (Thermo Scientific, Rockford, IL). Protein was digested 1 to 50 enzyme to protein with trypsin, chymotrypsin, and trypsin/Glu-C overnight at 37°C; note all enzymes purchased from Roche Diagnostics GMBH, Germany. For HSA-CEP and HSA-CTL1 digestions, 125 µg protein was denatured using ProteaseMAX surfactant (Promega, Madison, WI), reduced with 5 mM DTT, alkylated with 15 mM iodoacetamide and digested with 1 to 50 trypsin to protein overnight at 37°C.

#### Reverse phase LC-MS/MS analysis

Resulting peptides from MSA-CEP and MSA-CTL2 were analyzed by LC-ESI MS/MS on a Thermo Velos Orbitrap coupled to a Waters nanoACQUITY UPLC (Milford, MA). 70 pmol of digested MSA-CEP and MSA-CTL2 were HPLC separated on column (Waters Acquity HSS T3 1.8 µm beads, 1 × 100 mm at 40°C) at 15 µL/min. The 55 min gradient started 0–3 min, 3% B (B = acetonitrile, 0.1% formic acid), increased to 97% B at 35 min, then 95% B at 37 min, followed by washing and column equilibration. Mass spectrometer parameters included a full scan event using the FTMS analyzer at 100000 resolution from m/z 300–2000 for 10 ms. Collision induced dissociation (CID) MS/MS was conducted on the top seven intense ions (excluding 1+ ions) in the ion trap analyzer, activated at 500 (for first event) and 2000 (for remaining events) signal intensity for 10 ms.

For HSA-CEP and HSA-CTL1 digestion, resulting peptides were analyzed by LC-ESI MS/MS on a Thermo LTQ Orbitrap Discovery coupled to Agilent CapLC (Santa Clara, CA). 10 pmol of digested HSA-CEP and HSA-CTL1 were HPLC separated on column (Waters Acuity BEH C18, 1.7 µm, 1 × 100 mm column at 40°C) at 10 µL/min. The 80 min gradient started 0–1 min, 4% B, increased to 7% B at 1.1 min, 45% B at 55 min, then 95% B at 63 min, followed by washing and column equilibration. Mass spectrometer parameters included a full scan event using the FTMS analyzer at 30000 resolution from m/z 300–2000 for 30 ms. CID MS/MS was conducted on the top seven intense ions (excluding 1+ ions) in the ion trap analyzer, activated at 500 (for all events) signal intensity for 30 ms.

#### Data analysis and database searching

All mass spectra were processed in Qual Browser V 2.0.7 (Thermo Scientific). Mascot generic files (mgf) were generated with MS DeconTools (R.D. Smith Lab, PPNL) and searched using Mascot V2.3.01 (Matrix Science Inc., Boston, MA) database search against the SwissProt database, V57, with 513,877 sequences. Search parameters included: enzyme: semitrypsin, chymotrypsin, none, or trypsin/Glu-C, allowed up to two missed cleavages; fixed modification carbamidomethyl on cysteines (when applicable); variable modifications searched: Arg-CEP on arginines, Gln->pyro-Glu (at N-term glutamine), Lys-CEP on lysines, oxidation on methionine; peptide tolerance: ±25 ppm; MS/MS tolerance: ±0.6 Da. Sequence coverage and CEP modification assessments were evaluated on peptide scores with >95% confidence. High-scoring peptide ions were then selected for manual MS/MS analysis using Qual Browser.

### Structural Analyses of CEP Adducts

#### SDS-PAGE analysis

After boiled for 5 min, 5 µg of total protein mixed with 4X sample buffer (Invitrogen, cat. # NP0007) was loaded on 4–12% NuPAGE Bis-Tris gel (Invitrogen, Cat. # NP0321BOX) with NuPAGE MOPS running buffer (Invitrogen, Cat. # NP0001). SeeBlue Plus2 Protein Ladder (Invitrogen, cat. # LC5925) or BenchMark Protein Ladder (Invitrogen, Cat. # 10747-012) was used to estimate the protein size. Gel was stained with SimpleBlue SafeStain (Invitrogen, Cat. # LC6060) for overnight at 4°C and destained with HPLC water. The gel image was taken by Bio-Rad ChemiDoc XRS+ Imaging System.

#### Size exclusion chromatography (SEC)

Human Serum Albumin (HSA) samples (20 µg) were injected on Shodex KW-803 column with 1 mL/min flow rate, 20 mM Tris, 200 mM NaCl, 0.25 mM TCEP, 3 mM NaN3, pH 7.5 as mobile phase on Agilent 1200 HPLC. UV signal was recorded at 280 nm by Agilent 1260 DAD detector. Mouse Serum Albumin (MSA) samples (50 µg) were injected on a Large S200 Column with GE Superdex 200 10/300GL and at 500 µL/min flow rate, 150 mM NaCl and 0.02% NaN3 in Dulbecco’s PBS as mobile phase on Agilent 1260 BioInert HPLC. UV signal was measured at 280 nm by Wyatt TREOS/OptiLab Rex.

#### Circular dichroism (CD)

Protein samples were diluted in 10X diluted PBS (pH 7.4) to achieve similar concentration. Baseline was blanked by 10X diluted PBS (pH 7.4). The CD spectra (average of five scans) of protein samples were collected from 260 nm to 190 nm on a Jasco J-815 CD Spectrometer with 0.02-cm path length quartz cell at 10°C.

### In Vitro Assays

#### Cell-based TLR assays

HEK293 cells expressing TLR2 and NFKB luciferase reporter (gift from Novartis Vaccine, Siena, Italy) were seeded at 30000 per well the night before. HSA-CEP was added and incubated for either 6 hr or 24 hr. Supernatant was collected for IL-8 ELISA (R&D, cat# DY208). NFkB luciferase activity was assayed on remaining cells, using Bright-Glo (Promega, cat# E2610). HEK293 cells in a separate plate with the same treatment were used for cell viability measurement using CellTiter-Glo (Promega, cat# G7570), according to manufacturer’s instruction.

Thp1 (ATCC, cat# TIB-202) was primed with 0.5% DMSO for overnight, at 100,000/well, then incubate with HSA-CEP for 24 hr, with TLR ligands (Pam3CSK4, FSL1, R837 and R848 were all from Invivogen; LPS was purchased from Sigma) as controls. Supernatant was collected for IL-8 ELISA (R&D), and the remaining cells were used for cell viability measurement using CellTiter-Glo.

#### In vitro tube formation assay

The CellPlayer GFP Angiokit-96 by Essen BioScience (Ann Arbor, MI) was used to measure tube formation in vitro. Briefly, GFP-transfected HUVEC were co-cultured with human fibroblasts in a specially designed medium for 11 days in a 96-well format. Cells were treated on days 1, 2, 5, 7 and 9 with VEGF, TLR agonists, or CEP-adducted or control-treated proteins. Fluorescence measurements (IncuCyte, Essen BioScience) were taken kinetically every 12 hours for the duration of the experiment and average tube length (mm/mm^2^) was quantified on the last day of the experiment according to the manufacturer’s instructions. Control wells received media alone. Reagents were obtained from the following sources: Human VEGF 165– Peprotech; Pam3CSK4– InvivoGen; LPS -Sigma-Aldrich; Poly (I:C) –InVivoGen.

### In Vivo Assays

#### Animals

All animal experiments were approved by the Animal Care and Use Committee at the Novartis Institutes for Biomedical Research. Upon arrival at the vivarium, mice were acclimated for at least 4 days before any studies were initiated. The animals were fed standard laboratory chow and sterile water ad libitum. Genotyping was performed on genomic DNA obtained from tail snips by standard procedures. All mouse strains were genotyped for the *Crb1* gene, to determine if they carried the *rd8* mutation.

C57BL/6N mice were obtained from Taconic; the *rd8* mutation was present in these mice. C57BL/6J mice were obtained from Jackson; the *rd8* mutation was absent in these mice. *Myd88*-deficient mice lacking exons 2–5 were generated at Novartis Institutes for Biomedical Research. Mice were backcrossed to C57BL/6J mice for at least 10 generations; the *rd8* mutation was absent in these mice. Heterozygous breeding generated littermate pups of each genotype, identified by PCR genotyping. *Myd88* deficiency was also functionally confirmed by the *in vivo* retinal infiltration assay below: mutant vs. littermate *wt* mice with treated with either TLR2 agonists and with TLR4 agonists and the retinal infiltration was measured as described (not shown).

#### Laser-induced choroidal neovascularization (CNV)

CNV was induced by laser injury in age and sex matched on a) C57BL/6N mice and b) C57BL/6J mice. Two in vivo experiments were performed with each mouse strain. After pupil dilation with 1% cylate and 10% phenylephrine, the mice were anesthetized and the retinas were visualized with a slit lamp microscope and a cover slip. The laser (Iridex Oculight GLx 532 nm green laser) was applied at 3 locations with a successful laser shot inducing a vaporization bubble. Laser pulses are applied to both eye yielding 6 CNV area data points per mouse and with 10 mice per group yielding 60 CNV area data points per test condition. Immediately after laser 2.0 µl of test article was injected into the subretinal space of both eyes. A sclerotomy was first made with a 30 gauge needle, and then the test article was injected through the same incision with a 33 gauge blunt tipped needle and a 10 µl Hamilton syringe. Injections were visualized under a surgical microscope with direct observation of a small retinal detachment. 7 days post laser, mice were injected i.v. with a vascular label and then euthanized. Mouse eyes were fixed in 4% paraformaldehyde; RPE-choroid-scleral complexes were isolated and mounted on microscope slides. Fluorescent images of each laser-induced CNV were captured using a Axiocam MR3 camera on a Axio.Image M1 microscope (Zeiss). The CNV lesion sizes were quantified with Axiovision software (Version 4.5 Zeiss). Inter-group differences were analyzed with an ANOVA with a Dunnett's multiple comparison test on GraphPad Prism 6 for Windows software. Data was masked during image acquisition and data analysis.

Recombinant human VEGF165 (Peprotech), IgG2A (R&D, MAB006) and a proprietary anti-mouse VEGF antibody (4G3) were reconstituted in sterile saline (Hospira) to a concentration of 0.05, 0.5, 2.5 or 3.3 mg/ml respectively. 1.4 or 1.9 mg/ml of CEP-MSA and MSA-CTL2 (control 2, mouse serum albumin treated but not adducted) or the other reagents were injected in to the subretinal space on day 0 immediately after a laser as described. After the application of laser burns and subretinal injections of test reagents, antibiotic ointment (Tobramycin or Neomycin ophthalmic ointment depending on availability) was applied to both eyes. The anti-VEGF antibody, 4G3, is a mouse anti-VEGF IgG1 antibody. It binds to mouse VEGF with an EC50 of 0.047 nM in a sandwich ELISA and neutralizes mouse VEGF binding to human VEGFR-2 with an EC50 of 0.15 nM in a binding assay (ELISA MSD).

#### Corneal neovascularization (CoNV)

Acute CoNV was induced in 7- to 9- week old anesthetized mice by complete removal of the corneal epithelium with mechanical abrasion, as detailed [14]. At the end of the studies, mice were humanely euthanized and the area of CoNV was quantitated as described [14]. Animals were randomized prior to treatment and analysis was performed in a masked fashion. N = 5–10 mice/group.

In studies using *Myd88−/−* mice (**Figure 6**), male knockout (KO) and male wild-type littermate controls (WT) were abraded on day 0 and euthanized at the end of the study on day 21 for analysis. *Myd88*-deficient and littermate wild-type mice are on the C57BL6/J background and are described above.

For other CoNV studies (**Figure S3**), C57BL6/N mice were used. The *Crb1* gene product is expressed in the retina, but not in the cornea [22]. In the study presented in **Figure S3C**, animals (N = 10–12 mice/group) were injected i.p. with PBS (200 µl), control antibody (IgG1, 0.5 mg/kg) or anti-VEGF antibody (4G3, 0.5 mg/kg) on days 0, 3 and 5 post-abrasion and eyes were collected on day 6 for analysis.

#### In vivo TLR2-mediated retinal leukocyte infiltration

TLR2 ligand, Pam3CSK4, was purchased from Invivogen. Female C57BL/6N mice (7 weeks old, Taconic) were treated with either dipeptide-CEP (400 µg per animal, in PBS) or Pam3CSK4 (25 µg per animal, in PBS) via intraperitoneal injection. Control animals received an intraperitoneal injection of sterile PBS. Eight hours after injection, mice were euthanized. Eyes were enucleated and were fixed in 4% paraformaldehyde. For immunostaining, retinas were dissected out. Macrophages was stained using the F4/80-Alexa 488 conjugated antibody (AbD serotec, Oxford, UK). Neutrophils were stained using a biotinylated-Gr-1 antibody (San Diego, CA) and an Alexa Fluor 594 conjugated streptavidin secondary antibody (Molecular Probes, Eugene, OR). After retinas were flat mounted onto glass slides, fluorescent images were taken. And F4/80 and Gr-1 positive cells on the retina were counted using Zeiss AxioVision program.

## Supporting Information

Figure S1 **Confirmation of CEP Adduction By ^1^H-NMR and LC-MS/MS.** A) *Structure for Dipeptide-CEP*. B) *^1^H-NMR of Dipeptide-CEP*. The signature peaks for CEP, lysine, and glycine are indicated. The CEP peaks were not detected in the unadducted dipeptide (not shown). C) *LC-MS/MS of completely hydrolyzed MSA-CEP*. MSA-CEP was enzymatically hydrolyzed and processed for LC-MS/MS analysis. Only MSA-CEP showed a peak corresponding to lysine-CEP; untreated MSA-CTL1 (not shown) and treated but unadducted MSA-CTL2 (lower panel) did not have the CEP peak. D) *^1^H-NMR of completely hydrolyzed HSA-CEP*. The signature peaks for CEP, Tyr, and Phe are indicated. The resonances corresponding to CEP were absent in HSA-CTL1 and HSA-CTL2 (not shown).
(TIFF)

Figure S2 **Peptide Mapping of CEP Adduction by LC-MS/MS.** A) *LC-MS/MS Analysis of Trypsinized HSA-CEP*. LC-MS/MS of trypsin digested HSA-CEP showed sequence coverage of 65% where bold residues represent observed peptides. In HSA-CEP 14 sites of CEP adduction were identified by this analysis (shown as underlined amino acids). B) *LC-MS/MS Analysis of Trypsinized MSA-CEP*. MSA-CEP was digested with trypsin, chymotrypsin, and trypsin-gluC yielding a sequence coverage of 92% with bold residues representing observed peptides. In MSA-CEP 40 sites of CEP adduction were identified by this analysis (shown as underlined amino acids). The initial signal and propeptides are not observed in the mature, processed protein sequence for HSA and MSA and are shown as italicized residues.
(TIFF)

Figure S3 **CoNV Model is VEGF-Driven.** A) *Progression of Neovascularization.* Adult C57BL/6N mice (N = 5 animals/group) were subjected to corneal abrasion on Day 0 and dissected corneas were analyzed for neovascularization area at different timepoints after abrasion. Neovascularization area progressively increased and plateaued around 2 weeks after abrasion. Statistical analysis was performed using one-way ANOVA with Dunnet’s post-test, comparing each time point to Naïve. Only the statistically significant differences between groups are indicated. B) *Upregulation of VEGFA Transcript*. Total RNA was prepared from dissected corneas from naïve mice and or cornea-abraded mice that were euthanized on Day 1 and Day 6 post-abrasion as indicated (N = 5 to 6 animals/group). First-strand cDNA was generated using the High Capacity RNA-to-cDNA Master Mix (Applied Biosystems). Pre-amplification products were generated using the Taqman PreAmp Master Mix Kit (Applied Biosystems) and a pool of FAM-labelled Taqman assays on demand (Applied Biosystems). qPCR was performed on diluted pre-amplification products using the same Taqman assays on demand in qPCR singleplex reactions. Relative quantification (RQ) performed using ΔΔCt method and data presented as RQ median with error bars as RQ min and RQ max. *VEGFA*, *PECAM-1*(expressed by vascular endothelial cells), and *β-actin* mRNA expression was normalized by expression of *β-actin* gene and expressed relative to naive animals. Statistical analysis was performed using one-way ANOVA with Dunnett’s post-test. C) *VEGF Ab inhibits CoNV.* Adult C57BL/6N mice (N = 10–12 animals/group) were subjected to corneal abrasion on Day 0 and injected intraperitoneally with the reagents as indicated on Days 0, 3, and 5 post-abrasion. Reagents included PBS (vehicle), control IgG1 Ab, and anti-VEGF antibody (4G3). The antibodies were dosed at 0.5 mg/kg. On Day 6 the animals were euthanized and CoNV area was measured by fluorescence microscopy as described in Materials and Methods. Statistical analysis was performed using one-way ANOVA with Dunnett’s post-test.
(TIFF)

Table S1 **Synthetic CEP Adducts Generated.**
(DOC)

File S1 **Supplemental Data and Methodology of the Mouse Laser-Induced CNV Assay.**
(DOCX)
