# Supplementary figures and images for: Lack of Involvement of CEP Adducts in TLR Activation and in Angiogenesis
Source: PLoS One. 2014 Oct 24;9(10):e111472. doi: 10.1371/journal.pone.0111472 (PMC4208838; doi:10.1371/journal.pone.0111472)

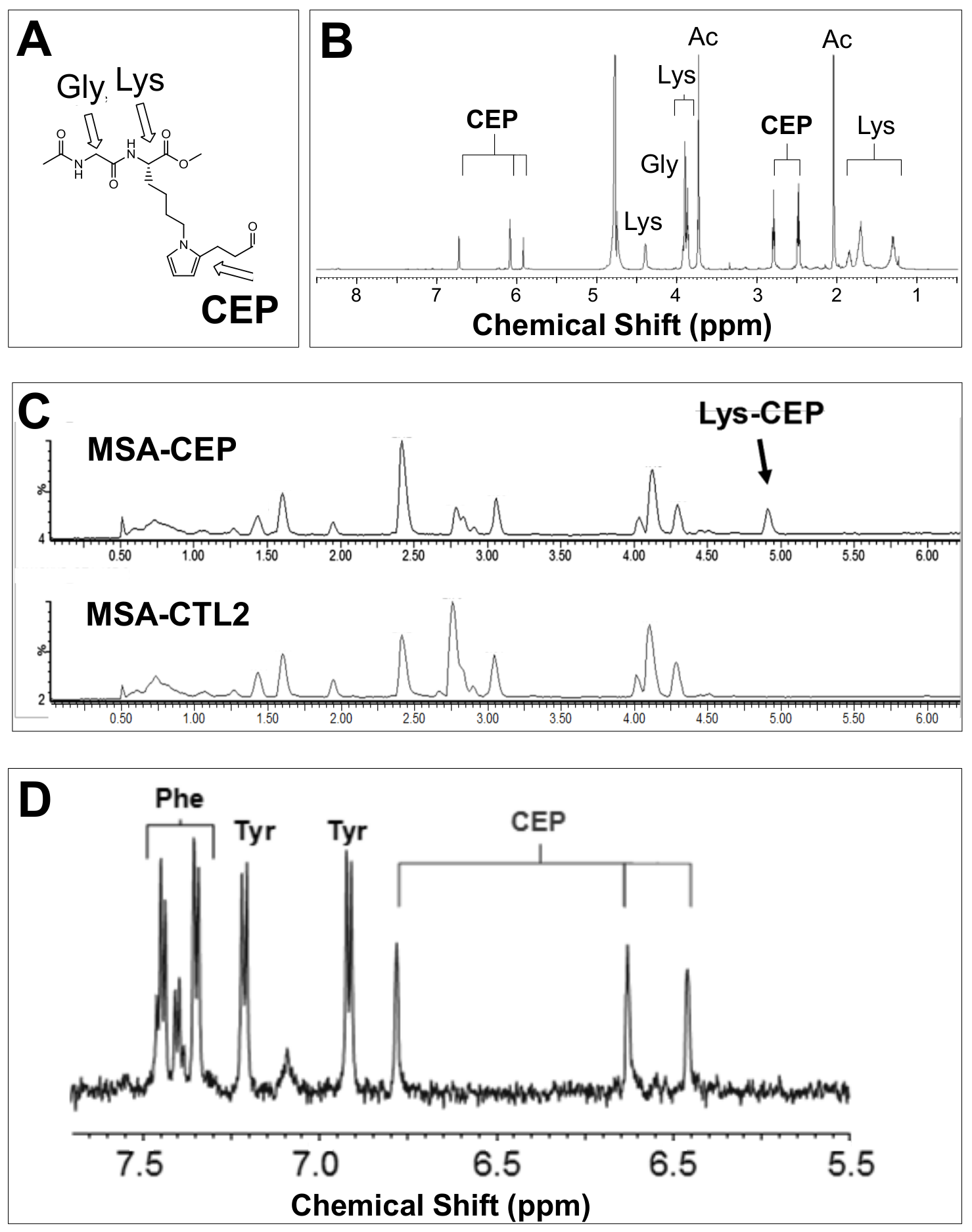

Supplement: Figure S1 — Confirmation of CEP Adduction By 1H-NMR and LC-MS/MS. A) Structure for Dipeptide-CEP. B) 1H-NMR of Dipeptide-CEP. The signature peaks for CEP, lysine, and glycine are indicated. The CEP peaks were not detected in the unadducted dipeptide (not shown). C) LC-MS/MS of completely hydrolyzed MSA-CEP. MSA-CEP was enzymatically hydrolyzed and processed for LC-MS/MS analysis. Only MSA-CEP showed a peak corresponding to lysine-CEP; untreated MSA-CTL1 (not shown) and treated but unadducted MSA-CTL2 (lower panel) did not have the CEP peak. D) 1H-NMR of completely hydrolyzed HSA-CEP. The signature peaks for CEP, Tyr, and Phe are indicated. The resonances corresponding to CEP were absent in HSA-CTL1 and HSA-CTL2 (not shown). (TIFF) [file pone.0111472.s001.tiff]

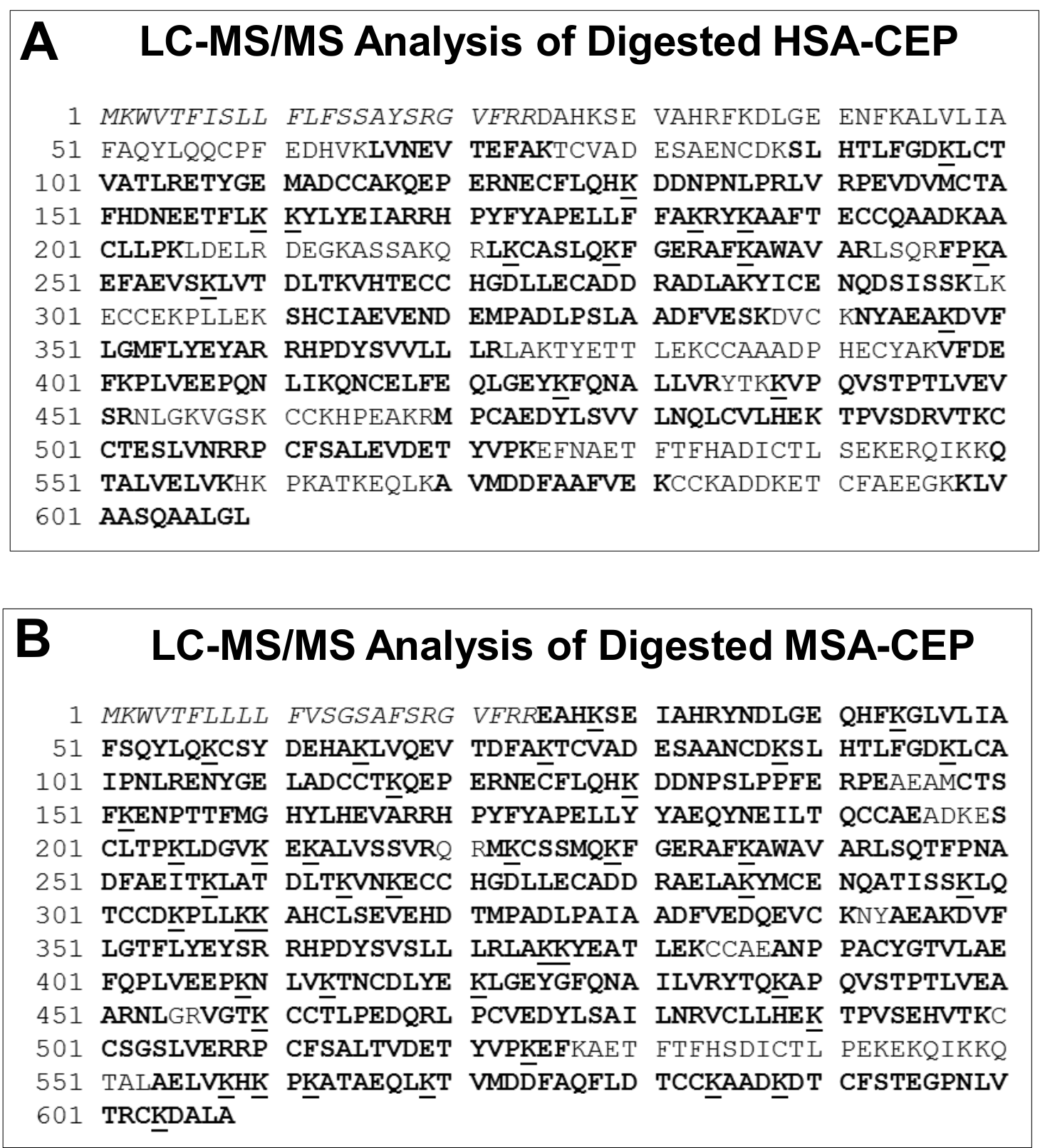

Supplement: Figure S2 — Peptide Mapping of CEP Adduction by LC-MS/MS. A) LC-MS/MS Analysis of Trypsinized HSA-CEP. LC-MS/MS of trypsin digested HSA-CEP showed sequence coverage of 65% where bold residues represent observed peptides. In HSA-CEP 14 sites of CEP adduction were identified by this analysis (shown as underlined amino acids). B) LC-MS/MS Analysis of Trypsinized MSA-CEP. MSA-CEP was digested with trypsin, chymotrypsin, and trypsin-gluC yielding a sequence coverage of 92% with bold residues representing observed peptides. In MSA-CEP 40 sites of CEP adduction were identified by this analysis (shown as underlined amino acids). The initial signal and propeptides are not observed in the mature, processed protein sequence for HSA and MSA and are shown as italicized residues. (TIFF) [file pone.0111472.s002.tiff]

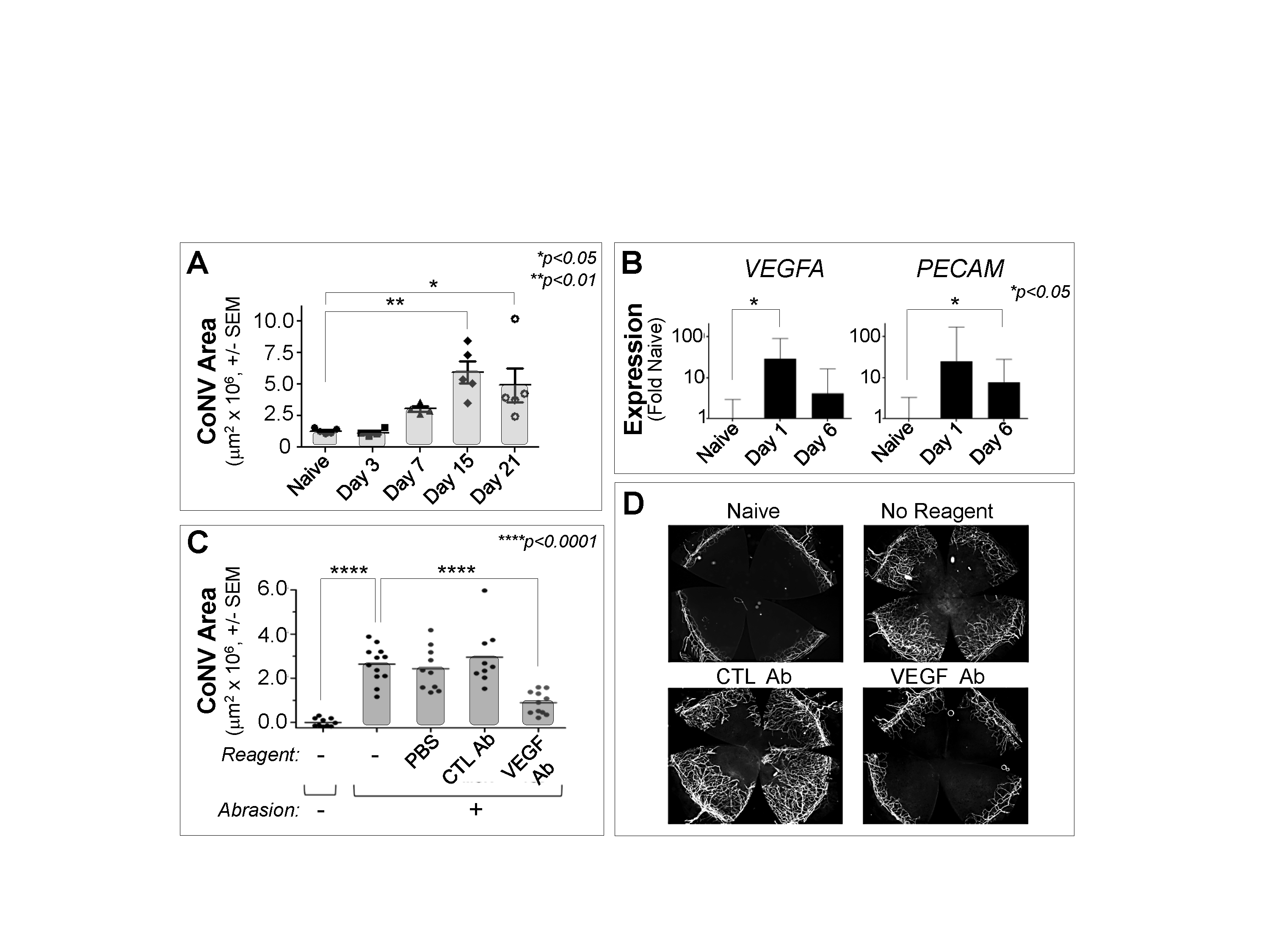

Supplement: Figure S3 — CoNV Model is VEGF-Driven. A) Progression of Neovascularization. Adult C57BL/6N mice (N = 5 animals/group) were subjected to corneal abrasion on Day 0 and dissected corneas were analyzed for neovascularization area at different timepoints after abrasion. Neovascularization area progressively increased and plateaued around 2 weeks after abrasion. Statistical analysis was performed using one-way ANOVA with Dunnet’s post-test, comparing each time point to Naïve. Only the statistically significant differences between groups are indicated. B) Upregulation of VEGFA Transcript. Total RNA was prepared from dissected corneas from naïve mice and or cornea-abraded mice that were euthanized on Day 1 and Day 6 post-abrasion as indicated (N = 5 to 6 animals/group). First-strand cDNA was generated using the High Capacity RNA-to-cDNA Master Mix (Applied Biosystems). Pre-amplification products were generated using the Taqman PreAmp Master Mix Kit (Applied Biosystems) and a pool of FAM-labelled Taqman assays on demand (Applied Biosystems). qPCR was performed on diluted pre-amplification products using the same Taqman assays on demand in qPCR singleplex reactions. Relative quantification (RQ) performed using ΔΔCt method and data presented as RQ median with error bars as RQ min and RQ max. VEGFA, PECAM-1(expressed by vascular endothelial cells), and β-actin mRNA expression was normalized by expression of β-actin gene and expressed relative to naive animals. Statistical analysis was performed using one-way ANOVA with Dunnett’s post-test. C) VEGF Ab inhibits CoNV. Adult C57BL/6N mice (N = 10–12 animals/group) were subjected to corneal abrasion on Day 0 and injected intraperitoneally with the reagents as indicated on Days 0, 3, and 5 post-abrasion. Reagents included PBS (vehicle), control IgG1 Ab, and anti-VEGF antibody (4G3). The antibodies were dosed at 0.5 mg/kg. On Day 6 the animals were euthanized and CoNV area was measured by fluorescence microscopy as described in Materials and Met [file pone.0111472.s003.tiff]
