# Supplementary material for: Lack of Involvement of CEP Adducts in TLR Activation and in Angiogenesis
Source: PLoS One. 2014 Oct 24;9(10):e111472. doi: 10.1371/journal.pone.0111472 (PMC4208838; doi:10.1371/journal.pone.0111472)
Supplement: Table S1 — Synthetic CEP Adducts Generated. (DOC) [file pone.0111472.s004.doc]

**Table S1. Synthetic CEP Adducts Generated**

Adduct Class MW^1^ (Da)

PE-CEP phospholipid 746

Dipeptide-CEP peptide 259

HSA-CEP protein 60,000

MSA-CEP protein 60,000

KLH-CEP protein ≥ 4000,000

Cytochrome C-CEP protein 12,000

Lysozyme-CEP protein 15,000

Myoglobin-CEP protein 17,000

All adducts were generated by the same procedure [10]. For HSA and MSA, treated unadducted controls (CTL2) were generated in parallel with the corresponding CEP adduct. PE = phosphatidyl ethanolamine; HSA = human serum albumin; MSA = mouse serum albumin; KLH = Keyhole limpet hemocyanin.

^1^ Molecular weight of untreated reagents, before adduction.
