# Supplementary material for: Lack of Involvement of CEP Adducts in TLR Activation and in Angiogenesis
Source: PLoS One. 2014 Oct 24;9(10):e111472. doi: 10.1371/journal.pone.0111472 (PMC4208838; doi:10.1371/journal.pone.0111472)
Supplement: File S1 — Supplemental Data and Methodology of the Mouse Laser-Induced CNV Assay. (DOCX) [file pone.0111472.s005.docx]

**Supplemental Data and Methodology of The Mouse Laser-Induced CNV Assay**

**Subretinal injection of MSA-CEP in mouse CNV assay, experiment #1**

(**A**) Bar graph shows mean area of CNV +/- SEM and (**B**) dot plot from individual data from first experiment evaluating the effect of subretinal injection of 5 µg of IgG2A, 0.5 µg rhVEGF_165_, 2.8 µg of MSA-CEP or 6.6 µg of 4G3 (an anti-mVEGF antibody) on laser-induced CNV in C57BL/6NTac mice from Taconic. The number above each bar is the percentage inhibition relative to average CNV area in mice injected with IgG2A. An additional table (C) is presented that contains information on data points that were included and excluded, mean CNV area, standard deviation, standard error, percentage inhibition and statistics (** p < 0.01, **** p < 0.0001)

(**A**)

 (B)





(C)

| Experiment 1 | IgG2A | MSA-CEP | hVEGF_165_ | VEGF Ab |
| --- | --- | --- | --- | --- |
| Dose (µg/eye) | 5 | 2.8 | 0.5 | 6.6 |
| Potential data points | 60 | 60 | 60 | 60 |
| CNV not measured | 1 | 1 | 3 | 7 |
| CNV lesions excluded | 9 | 13 | 20 | 18 |
| Number of CNV lesions used to calculate mean | 50 | 46 | 37 | 35 |
| Mean area CNV (mm^2^ x10^-3^ ) | 22.6 | 19.2 | 56.2 | 5.7 |
| Std. Deviation | 19.4 | 18.6 | 42.3 | 5.5 |
| Std. Error | 2.7 | 2.7 | 6.9 | 0.9 |
| % Inhibition | 0.0% | 15.0% | -149.4% | 74.7% |
| p value | NA | 0.84 | <0.0001 | 0.0064 |

**Subretinal injection of MSA-CEP in mouse CNV assay, experiment #2**

(**A**) Bar graph shows mean area of CNV +/- SEM and (**B**) dot plot from individual data from first experiment evaluating the effect of subretinal injection of saline, 0.1 or 0.5 µg of rhVEGF_165_, 2.8 µg of MSA-CEP or 6.6 µg of 4G3 (an anti-mVEGF antibody) on laser-induced CNV in C57BL/6NTac mice from Taconic. The number above each bar is the percentage inhibition relative to average CNV area in mice injected with IgG2A. An additional table (C) is presented that contains information on data points that were included and excluded, mean CNV area, standard deviation, standard error, percentage inhibition and statistics (** p < 0.01, **** p < 0.0001)





(A) (B)

(C)

| Experiment 2 | Saline | MSA-CEP | hVEGF | hVEGF | VEGF Ab |
| --- | --- | --- | --- | --- | --- |
| Dose (µg/eye) | NA | 2.8 | 0.1 | 0.5 | 6.6 |
| Potential data points | 60 | 60 | 60 | 60 | 60 |
| CNV not measured | 0 | 0 | 0 | 13 | 3 |
| CNV data points excluded | 13 | 11 | 15 | 26 | 14 |
| CNV data points used to calculate mean | 47 | 49 | 45 | 33 | 43 |
| Mean area CNV mm^2^ x10^-3^ | 22.4 | 26.0 | 48.6 | 49.5 | 7.39 |
| Std. Deviation | 19.3 | 26.3 | 43.1 | 28.7 | 7.0 |
| Std. Error | 2.82 | 3.75 | 6.43 | 5.00 | 1.07 |
| % Inhibition | 0.0% | -16.1% | 67% | -120.8% | 67.0% |
| p value | NA | 0.75 | 0.04 | <0.0001 | 0.004 |

**Subretinal injection of MSA-CEP in mouse CNV assay, experiment #3**

*The data for experiment #3 were shown in Figure 5. For clarity, the same data are shown here in the same format as that in the other laser CNV experiments in this SI.*

(**A**) Bar graph shows mean area of CNV +/- SEM and (**B**) dot plot from individual data from first experiment evaluating the effect of subretinal injection of saline, 0.5 µg of rhVEGF_165_, 3.8 µg of MSA-CTL2, 3.8 µg of MSA-CEP or 6.6 µg of 4G3 (an anti-mVEGF antibody) on laser-induced CNV in C57BL/6J mice from the Jackson Lab. The number above each bar is the percentage inhibition relative to average CNV area in mice injected with CTL2 (mouse serum albumin treated but not adducted with CEP). An additional table (C) is presented that contains information on data points that were included and excluded, mean CNV area, standard deviation, standard error, percentage inhibition and statistics (* p < 0.05, **** p < 0.0001)

(A) (B)







(C)

| Experiment 3 | CTL2 | Saline | CEP | hVEGF | VEGF Ab |
| --- | --- | --- | --- | --- | --- |
| Dose (µg/eye) | 3.8 | NA | 3.8 | 0.5 | 6.6 |
| Potential data points | 60 | 60 | 60 | 60 | 60 |
| CNV not measured | 8 | 6 | 1 | 0 | 7 |
| CNV data points excluded | 11 | 10 | 9 | 29 | 16 |
| CNV data points used to calculate mean | 41 | 44 | 50 | 31 | 37 |
| Mean area CNV mm^2^ x10^-3^ | 20.3 | 16.7 | 19.2 | 67.3 | 5.4 |
| Std. Deviation | 17.1 | 14.1 | 18.2 | 43.9 | 4.6 |
| Std. Error | 2.67 | 2.12 | 2.57 | 7.89 | 0.76 |
| % Inhibition | NA | **17.8%** | **5.4%** | **-231.5%** | **73.2%** |
| p value | NA | 0.86 | 0.99 | <0.0001 | 0.011 |

**Subretinal injection of MSA-CEP in mouse CNV assay, experiment #4**

(**A**) Bar graph shows mean area of CNV +/- SEM and (**B**) dot plot from individual data from first experiment evaluating the effect of subretinal injection of saline, 3.8 µg of MSA-CTL2, 3.8 µg of MSA-CEP or 6.6 µg of 4G3 (an anti-mVEGF antibody) on laser-induced CNV in C57BL/6J mice from the Jackson Lab. The number above each bar is the percentage inhibition relative to average CNV area in mice injected with CTL2 (mouse serum albumin treated but not adducted with CEP). An additional table (C) is presented that contains information on data points that were included and excluded, mean CNV area, standard deviation, standard error, percentage inhibition and statistics (* p < 0.05). Less than 20% of the data points in the group of mice injected with VEGF were assessable for analysis of CNV area, therefore this group was excluded from statistical analysis.

(A) (B)







(C)

| Experiment 4 | CTL2 | Saline | CEP | VEGF Ab | VEGF |
| --- | --- | --- | --- | --- | --- |
| Dose (µg/eye) | 3.8 | NA | 3.8 | 6.6 | 0.5 |
| Potential data points | 60 | 60 | 60 | 60 | 60 |
| CNV not measured | 2 | 1 | 1 | 2 | 0 |
| CNV data points excluded | 21 | 20 | 19 | 37 | 49 |
| CNV data points used to calculate mean | 37 | 39 | 40 | 21 | 11 |
| Mean area CNV mm^2^ x10^-3^ | 12.3 | 13.9 | 18.1 | 3.4 | 84.5 |
| Std. Deviation | 7.6 | 12.9 | 17.8 | 2.8 | 50 |
| Std. Error | 1.25 | 2.07 | 2.81 | 0.62 | 15.1 |
| % Inhibition | NA | **-13.2%** | **-47.0%** | **72.4%** | **-590%** |
| p value | NA | 0.89 | 0.11 | 0.03 | NA |

**Methods of laser-induced choroidal neovascularization in mice**

**Type, origin, and selection of mice**

C57BL/6NTac female mice were purchased from Taconic (Tarrytown, NY), and C57BL/6J female mice from The Jackson Laboratory (JAX, Bar Harbor, ME).  Mice used in a single experiment arrived at our research facility in a single shipment and were age-matched. The mice from Taconic were aged 8 or 10 weeks of age, the mice from Jackson Labs were aged 12 and 14 weeks for the four laser CNV experimental cohorts. Experimental interventions for a cohort of animals were started on the same day.  Laser application for an individual experiment was by a single scientist. Cages were randomly assigned to treatment groups with mice sharing a cage receiving the same treatment.

**Laser photocoagulation**

Mouse pupils were dilated with one drop (volume ~ 40 µl) of 1% cyclopentolate.  Just before anesthesia, pupil dilation was maximized with an additional drop of 10% phenylephrine.  Mice were then anaesthetized with an intraperitoneal (i.p.) injection of a mixture of ketamine and xylazine at doses of 80 - 100 mg/kg and 5 - 10 mg/kg, respectively. Prior to laser pulse application, each eye was anesthetized with topical 0.5% proparacaine.  Lubricating eyedrops (Genteal Alcon Laboratories) on a glass cover slip were applied to the cornea, and the retina was viewed through a slit lamp microscope.  Each laser pulse was applied approximately 0.5 to 1 mm from the optic nerve; single pulses in each of three separate locations were applied to each eye for a total of six laser photocoagulation sites for each mouse. The pulses were from a green laser (wavelength = 532 nm; Oculight, GLx, Iridex Corporation) and had a duration of 30 milliseconds, a power of 120 milliwatts, and a spot size of 100 microns.  A successful laser pulse generated a yellow vaporization bubble which correlated with a rupture of Bruch’s membrane (evaluated histologically in control mice sacrificed 6 hours after lasering; data not shown).  In cases when a vaporization bubble did not form (< 1% of laser pulses), one additional laser pulse could be administered to the same spot.  For each eye, a maximum of four laser pulses were allowed to generate 3 lesions.

**Compound administration**

Test articles injected by the subretinal route were administered immediately after laser pulse application. A sclerotomy was made with a 30 gauge needle about 1.5 mm below the limbus on the nasal side of the eye. A 33 gauge blunt-tipped needle attached to a 10 μl Hamilton syringe (Hamilton Company) was then inserted into the vitreous space through the sclerotomy and 2.0 µl of the test article was administered under direct visualization of a surgical microscope to the subretinal space on the temporal side of the eye. Approximately one quarter to one third of the retina is detached. The laser burns are distributed circumferentially around the optic nerve, in some cases, one burn may be overlapped by the detachment. Subretinal injections of test articles were administered bilaterally.

**Tissue processing, imaging and CNV area quantification**

Analysis of neovascularization was completed on tissues harvested 7 days after laser photocoagulation.  On the final study day, 0.1 ml of a 5 mg/ml solution of FITC concanavalin-A (Vector Laboratories) was injected intravenously (i.v.) to fluorescently label vascular endothelium.  Animals were euthanized 15 - 30 minutes later with inhaled carbon dioxide.  Eyes were enucleated and fixed in 4% paraformaldehyde (Vector Laboratories, Burlingame, CA) for approximately 60 minutes at room temperature, and then the fixative was replaced with PBS. Each eye was assigned a randomized number to mask the samples for the remainder of the analysis.  Posterior segments were isolated and retinas were removed.  The posterior eye cups which included the retinal pigment epithelium (RPE), the choroid, and the sclera, were flat-mounted onto microscope slides after making 3 or 4 radial cuts. Fluorescent images of each CNV lesion were photographed with an Axiocam MR3 camera on an Axio Image M1 microscope (Carl Zeiss Microscopy).  CNV area was quantified using a semi-automated analysis program (Axiovision software version 4.5, Carl Zeiss Microscopy) that outlined the fluorescent blood vessels.  Image capture, CNV area measurement and exclusions (see below) were performed on randomized samples or data by scientists masked to the treatment group.

**Application of exclusion criteria**

Each eye typically generates 3 data points corresponding to the area of 3 individual CNV lesions.  In a typical study, a cohort of 10 mice per group would optimally provide 60 data points.  However, a lesion would be excluded for any of the following reasons:  1) there was choroidal hemorrhage encroaching on the lesion; 2) the lesion was linear instead of circular, a consequence of a deflected (“split”) laser impact; 3) the lesion had fused with another lesion; 4) the lesion had a size indicating it was an outlier lesion as defined below; or 5) the lesion was the only lesion in an eye (i.e., if 2 of the 3 lesions in an eye were excluded, then all lesions in that eye were excluded).

An outlier lesion fell into one of the following three categories:  1) “too big”:  i.e., it was over 10,000 µ^2^ in area and was more than 5 times larger than the next biggest lesion in the eye (for reference, the mean area of CNV in a control group typically ranged from 10,000 to 20,000 µ^2^); 2) “too small”:  i.e., it was less than 1/5 the area of the next smallest lesion in the eye; this criterion applied only if at least one lesion in the eye was over 5,000 µ^2^; 3) “too different”:  i.e., after all of the lesions in a specific treatment group were measured, the lesion’s area was 5-fold greater than the mean for that group; this criterion applied only for lesions that were ≥ 5,000 µ^2^.

Other reasons that lesions were excluded or not measured include: 1) the death of a mouse before the end of an experiment; 2) the failure of the i.v. injection of the vascular label; 3) media opacities precluding accurate laser application (e.g., a pre-existing corneal scar or cataract); in this case the fellow eye could still be included; 4)  damage to the CNV lesion during tissue processing (i.e., poor quality of the tissue during processing so that the lesion could not be fully delineated); 5) inability to locate a CNV lesion during the imaging of an eyecup.

**Statistics**

One-Way ANOVA analysis was performed using Graph Pad Prism v6 (GraphPad Software, Inc.) for Windows with a Dunnett’s post hoc analysis test
